# Drivers of Routine Inpatient Laboratory Overuse and Preferred Stewardship Interventions Among Residents: A Cross-Sectional Survey at a Tertiary Care Center in Lebanon

**DOI:** 10.7759/cureus.109928

**Published:** 2026-05-30

**Authors:** Ali Ghais, Moied Al Sakan, Ouwais AlKhateb, Youssef Diab, Hadi Itani, Rose Daher, Ghazi Zaatari

**Affiliations:** 1 Internal Medicine, American University of Beirut, Beirut, LBN; 2 Cardiology, American University of Beirut, Beirut, LBN; 3 Internal medicine, American University of Beirut, Beirut, LBN; 4 Internal Medicine, University of Balamand, Beirut, LBN; 5 Internal Medicine, Donald and Barbara Zucker School of Medicine at Hofstra/Northwell, Hempstead, USA; 6 Laboratory Medicine, American University of Beirut, Beirut, LBN; 7 Pathology, American University of Beirut, Beirut, LBN

**Keywords:** education, hospital medicine, laboratory medicine, practice, residents

## Abstract

Objective: The objective of the study was to characterize residents’ self-reported inpatient laboratory and imaging ordering practices, perceived drivers of routine daily testing, and preferred stewardship strategies.

Methods: We conducted an anonymous cross-sectional web-based survey of internal medicine and general surgery residents at a tertiary academic medical center. The survey assessed resident characteristics, attitudes toward routine laboratory testing, self-reported ordering practices, perceived drivers of unnecessary testing, and preferred interventions to improve ordering behavior. Responses were summarized using counts and percentages, with Wilson 95% confidence intervals calculated for key proportions. Exploratory subgroup comparisons were performed using Fisher’s exact test.

Results: Among 115 invited residents, 62 complete responses were included (53.9%). Frequent routine laboratory ordering was reported by 40 (64.5%), although most respondents disagreed with daily laboratory testing irrespective of clinical need. The most commonly perceived unnecessary tests were chemistry panels, selected by 47 (75.8%), and complete blood counts, selected by 46 (74.2%). The most cited drivers of unnecessary testing were habitual practice, reported by 39 (62.9%), limited cost awareness, reported by 36 (58.1%), and anticipated attending requests, reported by 27 (43.5%). The most endorsed strategies were brief cost awareness sessions, selected by 37 (59.7%), bundled laboratory pricing, selected by 34 (54.8%), and removing daily laboratory options from order sets, selected by 27 (43.5%). Efficiency training on laboratory and imaging ordering was perceived as beneficial by 47 (75.8%). In exploratory subgroup analyses, frequent routine laboratory ordering was more common among junior than senior residents, 29 of 36 (80.6%) versus 11 of 26 (42.3%), Fisher’s exact p = 0.003, while no significant difference was observed between internal medicine and general surgery residents.

Conclusions: Residents perceived routine repeat inpatient laboratory testing as a low-value practice, yet many reported ordering routine tests frequently. The main perceived drivers were modifiable system and training factors, including habit, limited cost visibility, default order sets, and perceived attending expectations. These findings support the development of a resident-informed diagnostic stewardship intervention focused on practical education, cost transparency, and workflow changes.

## Introduction

Laboratory testing is central to inpatient care, supporting diagnosis, monitoring, and treatment decisions. Yet routine laboratory ordering in clinically stable inpatients is widely recognized as a form of low-value care and remains common across hospital settings [1]. National and international efforts have tried to standardize what “unnecessary daily labs” means in practice. Choosing Wisely recommendations specifically discourage repeating complete blood counts and basic chemistry panels in the face of clinical stability, emphasizing that tests should be ordered when they address a specific clinical question and are likely to change management [2]. This framing has helped shift the focus from individual test knowledge to ordering patterns and has motivated hospital-based diagnostic stewardship efforts aimed at reducing repetitive routine testing [3].

Even with clear recommendations, routine testing persists, with estimates around 20% reported in reviews [1]. Residents place many inpatient orders and are influenced by team norms, ordering defaults, and attending expectations [4,5]. Prior work shows they often order tests they view as unnecessary, commonly citing culture and limited cost transparency [6]. This makes resident-focused, local characterization important for identifying context-specific drivers and feasible targets for improvement. These issues are especially salient in Lebanon, where the health system has faced sustained strain and shortages in recent years, including disruptions in electricity and shortages of essential medicines [7]. While local stewardship work has been published for specific test initiatives, broader data describing routine inpatient laboratory ordering patterns among residents remains limited [8].

We aimed to describe residents’ attitudes and self-reported practices around routine inpatient laboratory and imaging orders and identify perceived drivers of low-value testing. We also explored resident-endorsed solutions to current practice. These findings will inform a locally feasible diagnostic stewardship bundle for quality improvement and future evaluation.

## Materials and methods

This was an anonymous cross-sectional web-based survey of internal medicine and general surgery residents at the American University of Beirut Medical Center, Beirut, Lebanon, from May 5, 2026, to May 14, 2026. Data were collected using an institution-hosted LimeSurvey platform (https://www.limesurvey.org/). The study was approved by the American University of Beirut Institutional Review Board (IRB number: SBS-2026-0125).

Participants

All current Internal Medicine and General Surgery residents who were still in training at the time of survey distribution were eligible and were invited via institutional email. There were no exclusion criteria based on age, sex, postgraduate year, or ethnicity. Ethnicity was not collected to preserve anonymity in a small single-institution resident cohort. Participation was voluntary and anonymous, and proceeding to the questionnaire implied consent. No incentives were offered. Incomplete survey responses were excluded from the analytic sample. 

Instrument

The questionnaire (see Appendices) was developed by the authors for this study and informed by prior literature [6]. It assessed resident characteristics, attitudes, and self-reported practices related to routine inpatient laboratory testing, perceived drivers of potentially unnecessary testing, and resident-supported solutions, including preferred stewardship strategies and the perceived value of efficiency training. Items on inpatient imaging were included for exploratory assessment. The draft survey was piloted with six residents for clarity and revised accordingly. In brief, the questionnaire included items on gender, age group, residency program, postgraduate year, attitudes toward daily laboratory testing, frequency of checking prior results and ordering routine tests, perceived unnecessary laboratory and imaging tests, cost awareness, perceived drivers of unnecessary testing, and preferred stewardship strategies.

Outcomes and variables

Main outcomes were resident attitudes, self-reported ordering practices, perceived drivers of unnecessary inpatient laboratory and imaging testing, and suggestions for stewardship interventions. Exploratory outcomes were perceptions of unnecessary inpatient imaging modalities. We also performed exploratory descriptive comparisons by training level (junior (Postgraduate Year (PGY)-1 to PGY-2) versus senior (PGY-3 to PGY-6)) and by specialty (internal medicine versus general surgery), presenting counts and percentages with confidence intervals (CIs) where appropriate. Exploratory subgroup comparisons were performed as described below. For multi-select items, respondents could select more than one option; therefore, percentages may sum to >100%.

In this study, ‘unnecessary’ refers to laboratory or imaging tests perceived as low-value when ordered routinely or repetitively in clinically stable inpatients and/or unlikely to change management, rather than appropriate targeted testing during initial diagnostic evaluation. This definition was not intended to refer to targeted testing during initial diagnostic evaluation, abnormal admission results, clinical deterioration, suspected complications, or other situations in which repeat testing is expected to guide management.

Data analysis

Survey data were exported from LimeSurvey and analyzed in Stata Statistical Software: Release 18 (StataCorp LLC, College Station, Texas, United States). We summarized responses as counts and percentages, reporting n and denominators for each item. For key proportions, we calculated Wilson 95% CIs. Likert scale responses were presented using prespecified category groupings, such as “always or most of the time” and “agree or disagree,” as appropriate. For multi-select items, percentages could exceed 100%. Missing data were not imputed, and denominators varied by item. Exploratory subgroup comparisons were performed using Fisher’s exact test. Two-sided p-values were reported, and analyses were considered exploratory. Free text responses were summarized descriptively. A two sided p value of <0.05 was considered statistically significant.

## Results

Participant characteristics

Of 115 eligible residents invited to participate, 62 complete responses were included in the analysis (response rate, 53.9%). Most were female, 33 (53.2%), and 35 (56.5%) were 26-30 years old. Internal medicine was the predominant specialty, comprising 48 (77.4%) participants. The largest group was PGY 1, with 19 (30.6%) participants (Table 1).

**Table 1:** Respondent characteristics (N=60) PGY: postgraduate year

Characteristics		Frequency (Percentage)
Sex	Female	33 (53.2%)
	Male	29 (46.8%)
Age	20–25	19 (30.6%)
	26–30	35 (56.5%)
	31–35	8 (12.9%)
Residency program	Internal medicine	48 (77.4%)
	General surgery	14 (22.6%)
Postgraduate year	PGY-1	19 (30.6%)
	PGY-2	17 (27.4%)
	PGY-3	12 (19.4%)
	PGY-4	7 (11.3%)
	PGY-5	3 (4.8%)
	PGY-6	4 (6.5%)

Attitudes toward routine laboratory testing

Most respondents disagreed with ordering daily labs irrespective of clinical need (n=47, 75.8%), while 10 (16.1%) agreed and five (8.1%) were unsure. The majority (n=53, 85.5%) believed not all daily lab orders are necessary.

Ordering Practices

Frequent routine laboratory ordering was reported by 40 (64.5%) residents. Most residents also reported checking prior laboratory results before ordering, either always (n=33, 53.2%) or most of the time (n=22, 35.5%).

Perceived Unnecessary Laboratory Testing

**Figure 1:**
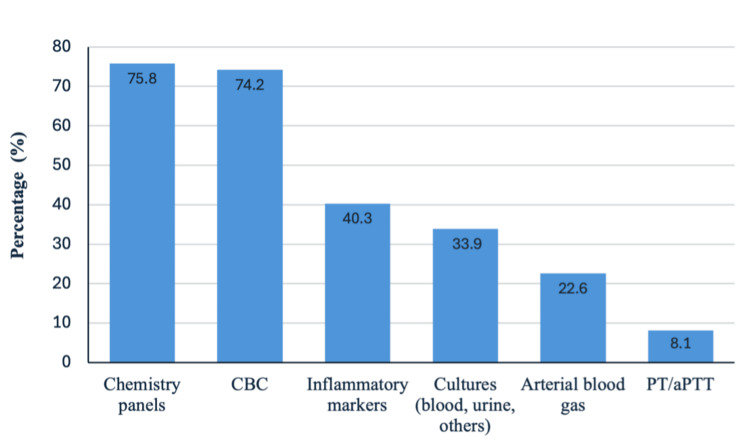
Laboratory tests perceived as overused by residents Data given as percentages PT: prothrombin time; aPTT: activated partial thromboplastin time

Most participants (n=60, 96.8%) believed certain routine laboratory tests are often ordered unnecessarily, particularly chemistry panels (n=47, 75.8%), complete blood counts (n=46, 74.2%), inflammatory markers (n=25, 40.3%), cultures (n=21, 33.9%), and arterial blood gases (14, 22.6%) (Figure 1).

Perceived Unnecessary Inpatient Imaging

**Figure 2:**
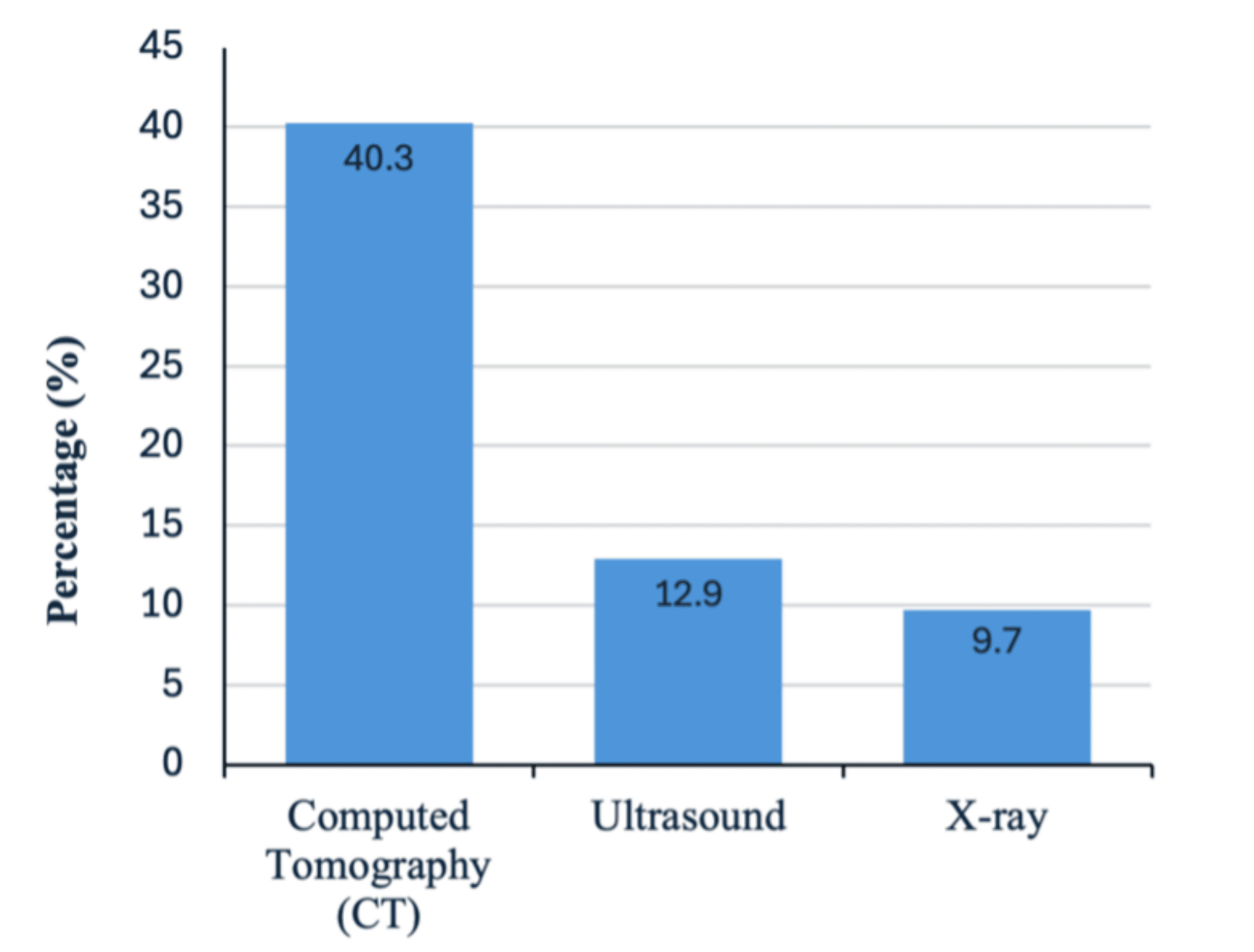
Imaging tests perceived as overused by residents Data given as percentages

More than three-quarters (n=48, 77.4%) believed some imaging is often unnecessary, most frequently CT, selected by 25 (40.3%), followed by ultrasound, selected by eight (12.9%), and X-ray, selected by six (9.7%). No respondents selected magnetic resonance imaging as "often unnecessary" (Figure 2).

Cost awareness

Just over half (n=34, 54.8%) of the respondents reported awareness of the cost burden of laboratory tests and imaging. Similarly, 34 (54.8%) took price into account when ordering, compared with 22 (35.5%) who did not and six (9.7%) who were unsure.

Contributing factors to unnecessary testing

Commonly cited drivers were habitual practice, reported by 39 (62.9%), lack of cost awareness, reported by 36 (58.1%), fear of being asked about results by attendings, reported by 27 (43.5%), and ease of ordering or repeat draws, reported by 25 (40.3%). Additional contributors included team expectations (n=22, 35.5%), diagnostic uncertainty, 19 (30.6%), fear of litigation, 18 (29.0%), and discomfort with not knowing results, 10 (16.1%).

Strategies to improve practice

Participants supported cost awareness sessions (n=37, 59.7%), bundled laboratory pricing (n=34, 54.8%), and removing daily laboratory options from order sets (n=27, 43.5%). Free text suggestions emphasized cost transparency, audit and feedback, and linking orders to clinical assessment, for example: ‘Educating residents and supervisors on test costs, including approximate prices’ and ‘Audit routine lab ordering and give case-based feedback.’ In addition, 47 (75.8%) residents believed efficiency training on laboratory and imaging ordering would be beneficial. Key outcomes with Wilson 95% confidence intervals are shown in Table 2.

**Table 2:** Key survey outcomes: practices, perceptions, drivers, and proposed strategies

Domain	Measure	Frequency (Percentage)	95% CI
Practices	Frequent routine lab ordering (“Always + Most of the time”)	40 (64.5%)	52.1–75.3
	Check previous results (“Always + Most of the time”)	55 (88.7%)	78.5–94.4
	Check results after ordering (“Always + Most of the time”)	57 (91.9%)	82.5–96.5
Perceptions	Any unnecessary laboratory testing perceived	60 (96.8%)	89.0–99.1
	Any unnecessary imaging perceived	48 (77.4%)	65.6–86.0
Drivers	Habitual practice	39 (62.9%)	50.5–73.8
	Lack of cost awareness	36 (58.1%)	45.7–69.5
Strategies	Efficiency training would be beneficial	47 (75.8%)	63.8–84.8
	Bundled lab pricing	34 (54.8%)	42.5–66.6
	Remove “daily labs” option	27 (43.5%)	31.9–55.9
	Five-minute cost-awareness session	37 (59.7%)	47.3–71.0

Exploratory subgroup comparisons

In exploratory subgroup analyses, frequent routine laboratory ordering was reported by 29 of 36 junior residents (80.6%) compared with 11 of 26 senior residents (42.3%) (Fisher’s exact p = 0.003). Other responses were not significantly different by seniority, including checking previous laboratory results before ordering and checking results after ordering. No statistically significant differences were observed between internal medicine and general surgery residents across the assessed ordering practices (Table 3).

**Table 3:** Exploratory subgroup comparisons of ordering practices P values were calculated using Fisher’s exact test; Junior residents were PGY 1 to PGY 2, and senior residents were PGY 3 to PGY 6. PGY: postgraduate year

Measure	Junior residents (n = 36), n (%)	Senior residents (n = 26), n (%)	p value	Internal medicine, (n = 48), n (%)	General surgery (n = 14), n (%)	p value
Believes all daily labs are necessary	2 (5.6%)	0 (0.0%)	0.505	1 (2.1%)	1 (7.1%)	0.403
Orders routine labs always or most of the time	29 (80.6%)	11 (42.3%)	0.003	30 (62.5%)	10 (71.4%)	0.752
Checks previous labs before ordering always or most of the time	32 (88.9%)	23 (88.5%)	1.000	43 (89.6%)	12 (85.7%)	0.651
Checks result after ordering always or most of the time	35 (97.2%)	22 (84.6%)	0.152	45 (93.8%)	12 (85.7%)	0.314

## Discussion

In this single-center survey of internal medicine and general surgery residents at a tertiary care center in Lebanon, most respondents were against ordering routine daily laboratory testing in clinically stable inpatients, yet nearly two-thirds reported doing so always or most of the time. Chemistry panels and complete blood counts were the tests most often perceived as overused, followed by inflammatory markers and cultures (Figure 1). Together, these findings suggest a discordance between attitudes and ordering behavior. This gap may be especially consequential in Lebanon, where out-of-pocket spending represents a substantial component of health expenditure and the health system has faced sustained operational strain [7,9]. In this setting, reducing low-value testing is not only a “value” issue but also a patient-burden and capacity-preservation issue.

In our cohort, residents most often pointed to habit, limited cost visibility, ease of repeat ordering, and attending expectations as drivers of routine testing. Similar dynamics have been described elsewhere, where clinicians report pressures such as malpractice concerns, perceived patient demand, and discomfort with uncertainty as drivers of overuse [10]. In hierarchical training settings, low psychological safety can discourage residents from questioning routine orders, reinforcing “just-in-case” repeat testing even when clinical stability makes the marginal benefit small [11]. This pattern suggests an environmental problem more than a knowledge problem.

Repetitive laboratory testing can also cause direct patient harm. In a resource-constrained inpatient setting, the cumulative burden of repeated blood draws and downstream investigations matters because capacity is limited and patients often carry direct costs. Repeated phlebotomy may contribute to iatrogenic anemia, disrupt sleep, fragment rest, and degrade the inpatient experience [12,13]. In addition, broad or repetitive testing can generate incidental or borderline abnormalities that trigger cascades of follow-up investigations with uncertain benefit, increasing burden and iatrogenic risk without proportionate clinical gain [14,15].

Imaging patterns in our survey suggest related workflow effects. CT was most often perceived as overused, whereas ultrasound and X-ray were selected less frequently, and no respondents selected MRI (Figure 2). This may reflect accessibility and ordering authority, as CT is commonly used as a rapid “rule-out” test, while MRI is more time-intensive and often gated by specialty approval, making it less likely to be initiated by residents. Even when immediate complications are uncommon, unnecessary CT exposes patients to ionizing radiation and can uncover incidental findings that trigger downstream tests and procedures with limited clinical benefit [16]. Because imaging items were exploratory, these findings are hypothesis-generating rather than definitive.

The training stage appears to be a practical target. Although both junior and senior residents largely disagreed with routine daily laboratory testing, junior residents reported significantly more frequent routine ordering than senior residents (Table 3). This may reflect lower confidence early in training, greater reliance on default workflows, closer adherence to unit norms, or increased concern about missing abnormalities. No statistically significant differences were observed by specialty, although the general surgery subgroup was smaller. These findings support prioritizing early training interventions, such as structured feedback on ordering patterns, senior-led role modeling, and team-level prompts to review prior results before reordering.

This approach is supported by evidence that cost display alone may not change overall ordering behavior [17], whereas multicomponent workflow and feedback strategies can be more reliable when tailored to local design and delivery [5,18]. A future resident-informed stewardship bundle could combine brief cost awareness teaching, daily review of whether repeat laboratory tests are needed, modification of default daily laboratory orders, and feedback on routine laboratory use. Faculty and senior resident involvement would be important to reinforce indication-based ordering during rounds. Routine laboratory tests per patient day could be used as the main outcome, with feasibility, acceptability, and adoption assessed during implementation.

Finally, this single-center cross-sectional survey has several limitations. The study was conducted in one tertiary care center in Lebanon, which may limit generalizability to other settings. The final analytic sample was limited by the small eligible resident population, voluntary participation, and exclusion of incomplete responses. Findings were self-reported using a non-validated instrument and may reflect local norms or varying interpretations of unnecessary testing. We did not collect ethnicity or patient-level ordering data, so we could not directly assess objective test appropriateness or clinical indications for ordering.

## Conclusions

In a resource-constrained medical setting, routine repeat inpatient laboratory testing can create an avoidable burden for both patients and hospitals. Residents largely disagreed with daily labs in clinically stable patients, yet many reported ordering them frequently, suggesting modifiable drivers such as habit, default order sets, limited cost visibility, and perceived expectations. Residents endorsed pragmatic solutions, particularly brief cost awareness teaching, bundled pricing, and removing daily lab defaults, and most felt efficiency training would help. These findings support a resident-informed stewardship bundle for implementation and evaluation using routine labs per patient day with prespecified balancing measures.

## Appendices

**Table 4:** Survey questionnaire administered to internal medicine and general surgery residents The questionnaire was developed by the authors for this study and informed by prior literature, including Sedrak et al. [6].

Section	Item	Question	Response options
General information	1	Gender	Male; Female; Prefer not to say
	2	Age	20 to 25; 26 to 30; 31 to 35
	3	Residency program	Internal medicine; General surgery
	4	Years in training	PGY 1; PGY 2; PGY 3; PGY 4; PGY 5; PGY 6
Views on the current state of medical laboratory testing	5	Do you support the practice of daily laboratory testing over necessity based testing?	Agree; Disagree; Unsure
	6	Do you believe that all laboratory tests performed on a daily basis are necessary for optimal patient care?	Agree; Disagree; Unsure
	7	On average, how often do you check previous laboratory results before requesting new ones?	Always; Most of the time; Sometimes; Rarely
	8	On average, how often do you order routine laboratory tests on a patient?	Always; Most of the time; Sometimes; Rarely
	9	On average, how often do you check the laboratory test results after ordering them?	Always; Most of the time; Sometimes; Rarely
	10	Which routine laboratory tests do you believe are more often ordered unnecessarily or redundantly? Select all that apply.	Complete blood count (CBC); Chemistry (eg, Chem 9 or others); Inflammatory markers (eg, CRP or others); Cultures (urine, blood, or others); Arterial blood gas (ABG); PT, aPTT; Other
	11	Which routine imaging tests do you believe are more often ordered unnecessarily or redundantly? Select all that apply.	X ray; Ultrasound; Computed tomography scan (CT scan); Magnetic resonance imaging (MRI); Other (specify)
Physician awareness	12	Are you aware of the cost burden of laboratory tests and imaging studies?	Yes; No; Unsure
	13	Do you factor in the cost burden when ordering laboratory tests or imaging studies?	Yes; No; Sometimes; Unsure
	14	Tick all the factors that you believe contribute to the habit of ordering unnecessary routine labs or images. Select all that apply.	Practice habit (I am trained to order daily labs or imaging); Lack of cost awareness of labs or imaging; Team expectations; Ease of ordering repeat labs; Discomfort with diagnostic uncertainty; Lack of cost-conscious culture at our institution; Discomfort with not knowing; Ordering labs is cheap and not harmful, why not?; Concern that the attending will ask for new results and I will not have them; Lack of experience; Fear of litigation from missed diagnosis related to labs or imaging
Solutions	15	In your opinion, which of the following strategies is the best to improve the practice of laboratory testing?	Remove daily labs option; Five-minute cost awareness session in educational activities; Bundled laboratory pricing; Other (please specify)
	16	Do you believe that you would benefit from efficiency training regarding ordering laboratory tests or imaging studies?	Yes; No
